# ﻿*Gomphonemavancampianum* sp. nov. (Gomphonemataceae, Bacillariophyceae), a new large *Gomphonema* species from Europe

**DOI:** 10.3897/phytokeys.244.122153

**Published:** 2024-07-01

**Authors:** Bart Van de Vijver, Margaux Pottiez, Rémy Chavaux

**Affiliations:** 1 Meise Botanic Garden, Research Department, Nieuwelaan 38, 1860 Meise, Belgium Meise Botanic Garden, Research Department Meise Belgium; 2 University of Antwerp, Department of Biology – ECOSPHERE, Universiteitsplein 1, B-2610 Wilrijk, Belgium University of Antwerp Wilrijk Belgium; 3 Direction régionale Auvergne-Rhône-Alpes, Office français de la biodiversité, Site de Lyon – 5, place Jules Ferry, 69006 Lyon, France Direction régionale Auvergne-Rhône-Alpes, Office français de la biodiversité Lyon France

**Keywords:** Europe, France, *Gomphonema*, morphology, new species

## Abstract

During a French biomonitoring survey of the lakes in the region Rhône-Méditerranée-Corse, a large, unknown *Gomphonema* taxon was observed in a lake in the vicinity of the City of Lyon (Département du Rhône, France), that could not be identified using the currently available literature. Detailed light and scanning electron microscopy investigations revealed the presence of two types of valves, one bearing a thick marginal crest and valves lacking the crest. Following comparison with similar, mostly tropical *Gomphonema* species, the unknown taxon is described as new: *Gomphonemavancampianum***sp. nov.** Discriminating features of the new species include the peculiar valve shape resembling a Chinese spoon, the broad upper valve part with acuminate tip, the presence of shallow depressions in the axial area, distinctly punctate striae and the occasional presence of the marginal crest. The new species was observed in several samples collected in an oligo- to mesotrophic, calcium-carbonate rich lake with a high ecological quality.

## ﻿Introduction

The genus *Gomphonema* is one of the dominant freshwater genera in European rivers and lakes (Levkov et al. 2016; Abarca et al. 2020). During the past twenty-five years, there has been a steady increase in the number of taxa, not in the least due to intensive taxonomic work by Erwin Reichardt revising several groups within the genus, such as the *G.dichotomum* group (Reichardt and Lange-Bertalot 1991), the *G.pumilum* group (Reichardt 1997), the *G.coronatum*/*acuminatum* group (Reichardt 1999), the *G.truncatum* group (Reichardt 2001) and the *G.gracile* group (Reichardt 2015a). In 2016, Levkov et al. (2016) published a monograph on the genus *Gomphonema* in Northern Macedonia describing 30 new species, solely based on morphological features. Abarca et al. (2020) questioned the use of valve outline when analysing the core group within the genus *Gomphonema* and concluded that the “Gomphonema*core group has been overdescribed due to the use of outline as the main criterion for species delimitation*” (Abarca et al. 2020, p. 1) as they observed large outline variabilities without an underlying genetic differentiation. Nevertheless, unknown *Gomphonema* species, often with restricted ecological preferences, are still observed in lakes and rivers in Europe.

During a French biomonitoring survey of the lakes in the region Rhône-Méditerranée-Corse (southern France), several populations of a large, unusual *Gomphonema* taxon were observed in samples collected from the artificial Lac du Drapeau (“*Flag lake*”) located in the vicinity of the French City of Lyon (Dépt. du Rhône, France). The largest populations seemed to be associated with submerged helophytes and aquatic plants. Despite a detailed morphological analysis, comparing the unknown taxon with all similar larger *Gomphonema* species worldwide, the taxon could not be identified. This contribution describes in detail the morphology of the new species highlighting its remarkable level of variability. Its morphology is compared with known species from the *Gomphonemaapiculatum*Ehrenberg (1843: 416) group and the *G.augur*Ehrenberg (1841: 211) (including *G.apicatum*Ehrenberg 1854: pl. 9, fig. 41) group. As a result of the morphological comparison, the new species is described as new: *Gomphonemavancampianum* Van de Vijver, Pottiez & Chavaux, sp. nov. Details on its ecology are added not only based on measured physicochemical parameters, but also derived from the accompanying diatom flora, integrating longer periods of ecological conditions.

## ﻿Materials and methods

Lac du Drapeau (elev. 170 m) is a 61 ha large lake of a calcium-carbonate sedimentary nature with a maximum depth of 3.2 m. The Lake was created following the extraction of materials in the Rhône plain and is fed by the aquifer of the Island of Miribel-Jonage and the Rizan Stream and finally flows into the Eaux Bleues gravel pit. Although the surrounding area experiences some anthropogenic (mainly recreational) pressure, the Lac du Drapeau is closed to the public as it is used for flood control and serves as a drinking water reservoir for the City of Lyon (Agence de l’Eau Rhône Méditerranée Corse 2021).

Six samples were collected from three different localities in the Lake. At each locality, one sample was gathered by scraping off five submerged stones, while a second sample was collected by squeezing five aquatic plants, keeping the water in small plastic vials. All samples were immediately fixed with ethanol on site.

All samples were prepared for LM and SEM observations following the method described in van der Werff (1955). Small amounts of each sample were cleaned by adding 37% hydrogen peroxide (H_2_O_2_) and heating to 80 °C for about 1 h, after which the reaction was completed by addition of saturated potassium permanganate (KMnO_4_). Following digestion and centrifugation (three times for 10 minutes at 3700× rpm), the resulting cleaned material was diluted with distilled water to avoid excessive concentrations of diatom valves on the slides. Cleaned diatom material was mounted in Naphrax (refraction index 1.73) and analysed using an Olympus BX53 microscope at 1000x magnification (N.A. 1.30), equipped with Differential Interference Contrast (Nomarski) optics and the Olympus UC30 Imaging System, connected to the cellSense Standard programme. As middle striae are often more spaced, underestimating the actual stria density, the stria density was determined by counting striae between the central area and the apices. For SEM analysis, part of the suspension was filtered through 5-μm Isopore™ polycarbonate membrane filters (Merck Millipore), pieces of which were affixed with conductive double-sided adhesive carbon-tabs to aluminium stubs after air–drying. Stubs were subsequently coated with a platinum layer of 15 nm and studied using a JEOL-JSM-7100F field emission scanning electron microscope at 2 kV and a working distance of 4 mm. Slides, samples and stubs are stored at the BR-collection (Meise Botanic Garden, Belgium). Plates were prepared using Photoshop CS5.

Terminology used in the description of the various structures of the siliceous cell wall is based on Ross et al. (1979, areola structure), Cox and Ross (1981, stria structure), Round et al. (1990, raphe structure) and Reichardt (1999, genus features for *Gomphonema*). The new species was compared with different *Gomphonema* taxa described from different locations worldwide (Reichardt 1995, 1999, 2001; Metzeltin and Lange-Bertalot 1998, 2007; Jahn and Kusber 2004; Kociolek 2011; Levkov et al. 2016).

**Figure 1.**
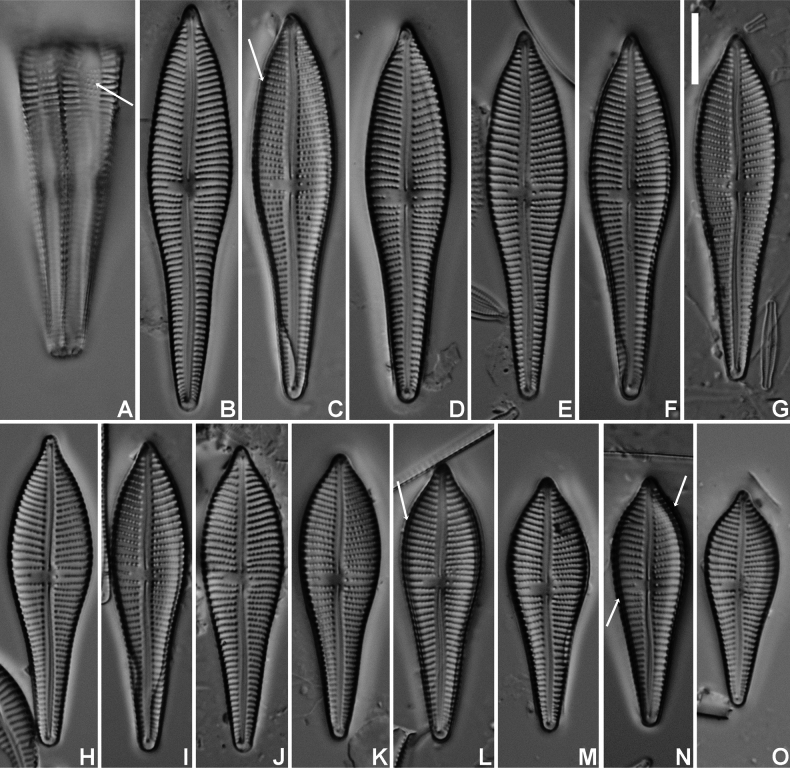
*Gomphonemavancampianum* sp. nov. LM micrographs taken from the holotype material (BR-4839, Lac du Drapeau, Sample DRAPEAU-U03VEG, France) **A** LM picture of a frustule in girdle view. The arrow indicates the smaller, more distantly spaced areolae on the mantle **B**–**O** LM pictures of valves in valve face view in decreasing length. The arrows indicate the possible presence of the marginal crest. Scale bar: 10 µm.

**Figure 2.**
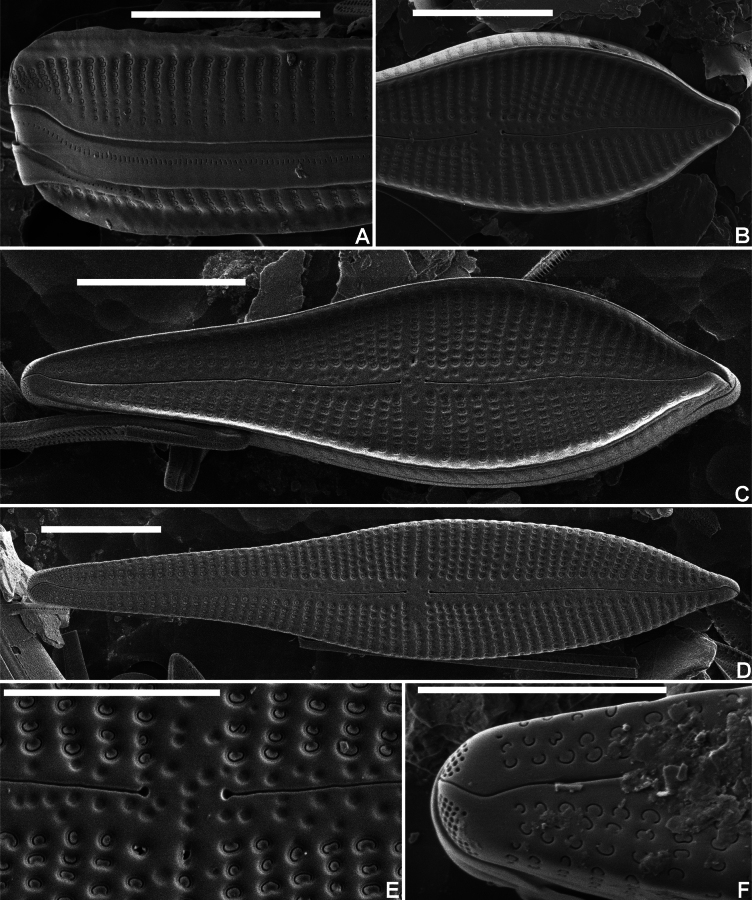
*Gomphonemavancampianum* sp. nov. SEM micrographs taken from the holotype material (BR-4839, Lac du Drapeau, Sample DRAPEAU-U03VEG, France) **A** SEM external view of the headpole in girdle view showing the marginal crest on both valves, the larger, reniformly occluded upper areolae and the smaller, more distant areolae towards the mantle edge. Note also the slit-like pores on the girdle band **B** SEM external detail of the upper valve part of a valve bearing a crest showing the lowering marginal crest at the apex **C** SEM external view of an entire crest-bearing valve. Note the shallow depressions in the axial area **D** SEM external view of valve without marginal crest. Note the shallow depressions in the axial area and the valve face striae continuing over the valve face/mantle junction **E** SEM external detail of the central area with the depressed large areolae and the stigmoid. The shallow pit-like depressions are well visible in the axial area **F** SEM external detail of the footpole with the distal raphe fissure bisecting the apical pore field. Scale bars: 10 µm (**A–D**), 5 µm (**E–F**).

**Figure 3.**
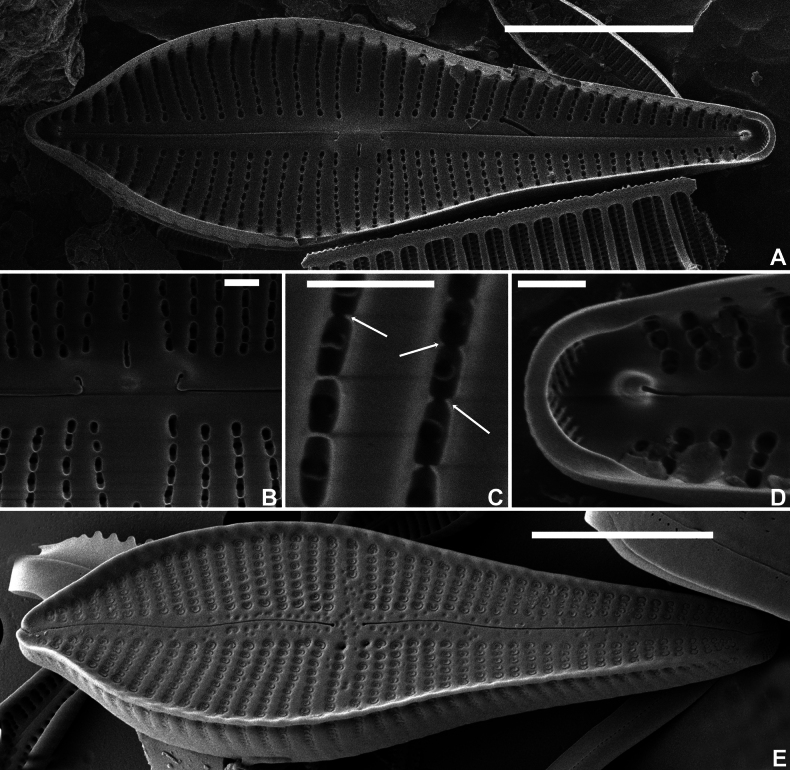
*Gomphonemavancampianum* sp. nov. SEM micrographs taken from the holotype material (BR-4839, Lac du Drapeau, Sample DRAPEAU-U03VEG, France) **A** SEM internal view of an entire valve **B** SEM internal detail of the central area with the stigmoid located in a long depression, the hooked central raphe endings and the long foramina with the areolae **C** SEM internal detail of the areolae with the small silica struts **D** SEM internal detail of the footpole with the small pseudoseptum, the helictoglossa and the apical pore field **E** SEM external view of an entire valve with marginal crest from Lac du Réaltor, Provence, France (photo courtesy of Dr Carlos E. Wetzel). Scale bars: 10 µm (**A, E**), 1 µm (**B–D**).

For typification of the species, we chose to use the entire slide as the type, following article 8.2 of the International Code for Botanical Nomenclature (Turland et al. 2018). Diatoms show a broad variability during their cell cycle and choosing the entire population present on a slide as the type shows this variability. One valve was indicated to illustrate a typical valve of the new species (see Figs 1–3) to avoid confusion with other *Gomphonema* taxa. All novelties are registered proactively according to Art. 42.3 (Turland et al. 2018).

## ﻿Results

### ﻿ Gomphonema vancampianum

Taxon classificationPlantaeCymbellalesGomphonemataceae

Van de Vijver, Pottiez & Chavaux sp. nov.

A0870673-631B-5C1A-98DE-066D3CB047DB

Figs 1 , 2 , 3

#### Type materials.

***Holotype*.** BR-4839 (Meise Botanic Garden, Belgium). Fig. 1C represents the holotype. ***Isotype*.** Slide 441 (University of Antwerp, Belgium).

#### Registration.

http://phycobank.org/104517.

#### Type locality.

Lac du Drapeau (Lyon, Département du Rhône, France), sample Drapeau-U03-VEG, (coll. date 22.viii.2023, leg. R. Chavaux).

#### Etymology.

This species is named in honour of Prof. Dr Karel Van Camp, former Physics professor of the first author at Antwerp University (Belgium) and life-long enthusiastic amateur diatomist and microscopist.

#### Description.

***LM*** (Fig. 1). Frustules in girdle view narrowly clavate with transapical striae continuing on to the valve mantle without interruption; adjacent to the striae, a line of isolated areolae (Fig. 1A, arrow). Headpole much broader than the footpole. Valves apiculate-clavate with elliptic-lanceolate upper valve part and largest width right above the valve middle. Lower valve part abruptly narrowing near the valve centre, then gradually tapering towards the acute footpole. Headpole acutely rounded with a narrow protracted, cuneate apex. Occasionally, valves surrounded by marginal crest, visible by changing focal depth (Fig. 1C, L, N, arrows). Valve dimensions (n = 25): length 30–60 µm, width 10–12 µm. Axial area moderately broad, linear with distinct, shallow markings, visible by changing focal depth. Central area asymmetrical: primary side with more distantly spaced single long stria and stigmoid, well separated near the valve middle. Isolated stria on the secondary side markedly shortened. Raphe clearly lateral and weakly undulating. Central raphe endings indistinct, almost straight. Terminal raphe fissures not discernible in LM. Striae parallel in the middle, soon becoming radiate towards the headpole, but remaining almost parallel or slightly radiate towards the footpole, 10–11 in 10 µm, more closely spaced near the apices. Striae distinctly punctate, 18–24 areolae in 10 µm. ***SEM*** (Figs 2, 3). Two types of valves present: with marginal crest (Fig. 2A–C) and lacking marginal crest (Fig. 2D, F). Valve face and mantle striae in crested valves interrupted at the valve face/mantle junction by the thickened marginal crest. Marginal crest with undulating border near the headpole (Fig. 2A). Mantle striae in advalvar part composed of large, densely packed, c-shaped areolae, externally covered by small reniform siliceous flaps. Areolae in abvalvar part of the striae towards the mantle edge, smaller, c- to e-shaped (Fig. 2A). Girdle bands broad with continuous row of transapically elongated narrow pores. Marginal crest lowering towards the apices (Fig. 2B, C). Valves lacking crest with striae extending almost continuously across the valve face/mantle junction (Fig. 2F). Axial area covered with dense irregular pattern of shallow, pit-like depressions (Fig. 2B, D, E). Raphe branches clearly undulating (Fig. 2C, D). Central raphe endings almost straight, small drop-like (Fig. 2E). Distal raphe fissure at footpole bisecting apical pore field, continuing on to the mantle (Fig. 2F), at headpole splitting the marginal crest (when present) and continuing shortly on to the mantle (Fig. 2C). Apical pore field bisected, composed of several rows of densely packed, rounded pores (Fig. 2F), only present at footpole. Striae composed of c-shaped areolae, occluded by small reniform siliceous flaps, near the central area slightly sunken into valve surface (Fig. 2E), towards the apices on the same level as the virgae (Fig. 2F). Stigmoid external opening small, rounded, sunken into valve face (Fig. 2E). Internally valve face surface smooth (Fig. 3A). Small pseudoseptum visible at footpole (Fig. 3A, D). Striae located in shallow, narrow foraminal rows. Side walls of the areolae with continuous apical bars or small interrupted struts (Fig. 3C, arrows). Internal opening of the stigmoid short, transversely elongated, located in a long, shallow groove (Fig. 3B). Central raphe endings long, right-angled, hook-shaped (Fig. 3B). Terminal raphe endings terminating on to well-developed helictoglossae (Fig. 3D).

#### Distribution and ecology.

*Gomphonemavancampianum* has so far only been found in several samples from the type locality in France. The largest population was found in a sample collected from submerged aquatic plants. The diatom flora is dominated by species typically found in calcium-carbonate rich, oligotrophic lakes with low nutrient concentrations and low saprobity. The sample is dominated by several cymbelloid taxa (e.g. *Encyonopsissubminuta* Krammer & E.Reichardt, *Cymbellaaffiniformis* Krammer, *C.lange-bertalotii* Krammer, *Cymbopleuraamphicephala* (Nägeli) Krammer) together with a, so far, unidentified, long-celled *Fragilaria* species, *Brachysiraneoexilis* Lange-Bertalot, B.cf.chiaruccii Cantonati et al. and Nitzschiacf.subacicularis Hustedt, all indicating the environmental conditions mentioned above (Lange-Bertalot et al. 2017). The species has also been found in several other French lakes, such as Lac du Réaltor (Provence, France) (Fig. 3E) by Luc Ector and Carlos E. Wetzel (LIST Luxemburg) who, despite an exhaustive review of existing *Gomphonema* literature, could not assign a name to this species (Wetzel, pers. comm.). It is likely that the species is more abundant than currently known due to confusion with similar taxa, such as *G.jadwigiae* Lange-Bertalot & E.Reichardt and *G.turris* Ehrenberg (see Discussion below).

## ﻿Discussion

Despite its rather large valve dimensions, its conspicuous form and structure, *Gomphonemavancampianum* could not be identified using all currently available literature. In the editorial note to Levkov et al. (2016, p. 1), Horst Lange-Bertalot wrote that “*Macedonia hosts approximately 70%, i.e. 125 of about 180*, Gomphonema*taxa currently known from all over Europe*”. A thorough comparison of all *Gomphonema* species illustrated in Levkov et al. (2016) did, however, not show any similar species. Cleve-Euler (1955), often a valuable resource for unusual diatom taxa, did not report any taxon showing the same peculiar valve outline, resembling a Chinese spoon with an acute headpole. Patrick and Reimer (1975, plate 15, fig. 1) illustrated one valve that most likely is conspecific with *G.vancampianum*. The valve was identified as *Gomphonemaapicatum* Ehrenberg and the drawing was based on a Boyer sample from Birges Pond, Connecticut (USA). The American population had a valve length of 45–50 µm and width of 13–14 µm. On the Diatoms of North America website, Kociolek (2011) showed several valves he identified as *G.apicatum*, probably basing his identification on Patrick and Reimer (1975), but the depicted valves differ from *G.vancampianum*. The description of *G.apicatum* is based on only two illustrations Ehrenberg (1854) published in his Mikrogeologie and, in fact, replace a species, previously named *G.augur* Ehrenberg. One of the illustrations (Ehrenberg 1854, plate IX, I, fig. 41a, b) consists of two drawings made of specimens from Ceyssat, France. These drawings originally had been labelled *Gomphonemaaugur* on the drawing sheet 2311, kept in the Ehrenberg collection, an additional indication that *G.apicatum* is, in fact, a superfluous name for *G.augur*. Jahn and Kusber (2004) lectotypified *G.augur*, based on material from Ceyssat, as was already suggested by Metzeltin and Lange-Bertalot (1998, p. 112). Despite being illegitimate, the name *G.apicatum* appeared in several historic diatom monographs. Cleve (1891, pp 48–49, plate III, figs 20–21) discussed *G.apicatum* and illustrated two valves whose outline resembles more *G.vancampianum* than *G.augur*. However, the valve dimensions of the specimens illustrated by Cleve are much lower (approximately half the size of *G.vancampianum*) than what was measured in all populations of *G.vancampianum* [length 22–25 µm, width 6–7 µm in Cleve (1891) versus length 30–60 µm, width 10–12 µm in *G.vancampianum*]. Most likely Cleve (1891) had illustrated *G.jadwigiae*, a species that was described from the famous Julma Öllky Lake in Finland (length 24–46 µm, width 5–7.5 µm) by Metzeltin & Lange-Bertalot in 1996. Cleve (1891) also referred to a species described by Ralfs (1843) as *G.cristatum* Ralfs that Smith (1853, p. 79) considered to be possibly a synonym of *G.augur* Ehrenberg as illustrated by Kützing (1844, plate XXIX, fig. 74). The drawings in Ralfs (1843, fig. 6) and Smith (1853, fig. 239) are indeed very similar to the lectotype of *G.augur*, illustrated in Jahn and Kusber (2004, figs 25–26). The Finnish specimen (“*aus demselben finnischen Gewässer*”), illustrated and discussed in Hustedt (1930, p. 372, fig. 696) as *G.apicatum*, most likely also represents *G.jadwigiae*, as the reported valve dimensions fit the latter (length 20–35 µm, width 6–9 µm). The same applies to Mayer (1928, p. 19) who discussed *G.apicatum*, but the drawings (Mayer 1928, plate 2, figs 16–17) and the valve dimensions (length 21–32 µm, width 6–7 µm) indicate that he most likely also refers to *G.jadwigiae*. None of these reported specimens, however, are conspecific with the species identified and discussed in Patrick and Reimer (1975) as *G.apicatum*.

Although similar in some respect (valve length, stria density, areolae discernible in LM), the North-American population, (erroneously) identified by Kociolek (2011) as *G.apicatum*, differs from *G.vancampianum*. The largest valve width in *G.apicatum* sensu Kociolek (2011) is positioned more closely to the headpole than in *G.vancampianum*. Kociolek (2011) also reported a higher valve width (13–15 µm) than measured for *G.vancampianum* (10–12 µm). Moreover, the apices in *G.vancampianum* have less developed shoulders in comparison with *G.apicatum*. The final tip on the apices in *G.apicatum* is more acute and longer than in *G.vancampianum*. The valves in *G.apicatum* gradually narrow towards the footpole, but in a straighter line than in *G.vancampianum*. It is unfortunate that the North-American population was not studied in SEM to compare the ultrastructure, which would have enabled a more thorough comparison. Finally, the shortened stria opposite the stigmoid in the central area, is always longer in *G.vancampianum*, compared to the illustrated valves of *G.apicatum*. Given the differences in valve outline and valve width and, despite the plea in Abarca et al. (2020) to use molecular evidence to support a possible differentiation of two species based on outline, we consider that there are sufficient morphological differences between both taxa to justify the description of *G.vancampianum* as a separate species.

*Gomphonemavancampianum* also shows some resemblance to a group of tropical species related to *G.apiculatum* Ehrenberg. De Toni (1891) considered this species as a synonym of *G.augur*, but the valves from the type material shown in Reichardt (1995) show clear differences from *G.augur* to exclude a possible conspecificity. *Gomphonemaapiculatum* may be conspecific with *G.vancampianum*, but with only one complete and one half specimen known and the drawing in Ehrenberg’s Mikrogeologie (Ehrenberg 1854, plate IV, II, fig. 39), it is almost impossible to come to a conclusion. The general valve outline in *G.apiculatum* is different showing more compact valves with only very gradually tapering margins towards the footpole, contrary to *G.vancampianum* that has a very narrow lower part of the valve. *Gomphonemaneoapiculatum* Lange-Bertalot, E.Reichardt & Metzeltin, was described in 1988 from the Essequibo River in Guyana (Metzeltin and Lange-Bertalot 1998). Metzeltin and Lange-Bertalot (1998, p. 120) stated in their discussion that it is unclear if *G.apiculatum* was validly described by Ehrenberg (1843) since the name *G.apiculatum* was put between brackets and replaced by the name ‘*G.augur*’. As Reichardt had illustrated 1.5 valves, identified as *G.apiculatum* from Ehrenberg’s Cayenne (Guyana) material, Metzeltin and Lange-Bertalot (1998) described these valves as *G.neoapiculatum*, a species widely distributed in the Neotropics. The valves illustrated in Metzeltin and Lange-Bertalot (1998, plate 157, figs 6–9) have a distinctly different valve outline with very gradually tapering margins and a less inflated upper part of the valve, excluding conspecificity with *G.vancampianum*. In 2007, Metzeltin and Lange-Bertalot described another species in this complex, *G.perapicatum* Metzeltin & Lange-Bertalot, but this species also lacks the concave lower part of the valve and the inflated upper part, making it sufficiently different from *G.vancampianum* to be the same species. Both *G.perapicatum* and *G.neoapiculatum* have a very elongated, acutely ending upper valve part, which has never been observed in *G.vancampianum*.

A final species showing some resemblance is *Gomphonematurris* Ehrenberg, described in 1843 from North America. The taxonomic history of the species had been analysed by Krammer and Lange-Bertalot (1985) who concluded that it should be considered only a variety of *G.augur* and subsequently made the new combination G.augurvar.turris (Ehrenberg) Lange-Bertalot. They illustrated the species with several pictures from a population from Manaus (Brazil), unfortunately not the type population (Krammer and Lange-Bertalot 1985, plate 37, figs 1–7) and a population based on a historic slide from the Grunow collection from Rio de Janeiro (Krammer and Lange-Bertalot 1985, plate 37, figs 1–4). Reichardt (2015b) tried to locate the type specimens Ehrenberg (1843) used for his new species. Unfortunately, since the material from West Point (New York) and Smithfield, proved to be devoid of *G.turris* following the analysis by Regine Jahn (Reichardt 2015b, p. 147), Reichardt (2015b) illustrated one valve from a Japanese population, as Ehrenberg (1854) had referred in his Mikrogeologie to both the American and the Japanese population. The latter, however, cannot be considered type material. The morphology of the type of *G.turris* is thus unclear. Based on the illustrations in Krammer and Lange-Bertalot (1985) and the Japanese specimen from the Ehrenberg sample in Reichardt (2015b), it is clear that *G.vancampianum* is not conspecific. *Gomphonematurris* has a complete different valve outline with a clearly undulating upper part, the presence of well-developed shoulders and a distinct apiculate headpole. The valves only gradually taper from the central area towards the footpole, contrary to *G.vancampianum* where there is an abrupt narrowing of the valve width below the central area before tapering to the footpole. Bahls (2023) illustrated a population he considered being *G.turris*, but these valves may represent *G.vancampianum* as they are in clear contrast with the generally accepted idea of *G.turris*, especially when considering the Japanese specimen in Reichardt (2015b). However, the valves in Bahls (2023) present an additional narrowing of the valve near the headpole contrary to the smooth, gradual margin in *G.vancampianum*, adding doubt to the possible conspecificity. It would be a good idea to analyse *G.turris* sensu Bahls (2023) in SEM to verify whether these valves also show the typical marginal crest. It is also likely that, in the illustrated valves in Bahls (2023, plate 52), several distinct species are included, but further analysis of these populations will be necessary to clarify this. An additional difference between the generally accepted idea of *G.turris* and *G.vancampianum* is the valve width: 12–20 µm in *G.turris* versus 10–12 µm in *G.vancampianum*, although the valve length overlaps. Therefore, conspecificity with *G.turris* should at present be excluded.

## Supplementary Material

XML Treatment for Gomphonema vancampianum

## ﻿Additional information

### Author ORCIDs

Bart Van de Vijver https://orcid.org/0000-0002-6244-1886

Margaux Pottiez https://orcid.org/0009-0009-2282-8355

### Data availability

All of the data that support the findings of this study are available in the main text.
